# Do Bolivian small holder farmers improve and retain knowledge to reduce occupational pesticide poisonings after training on Integrated Pest Management?

**DOI:** 10.1186/1476-069X-13-75

**Published:** 2014-10-01

**Authors:** Erik Jørs, Flemming Lander, Omar Huici, Rafael Cervantes Morant, Gabriel Gulis, Flemming Konradsen

**Affiliations:** Department of Occupational and Environmental Medicine, Odense University Hospital, and University of Southern Denmark, Odense, Denmark; Department of Occupational and Environmental Medicine, Herning Regional Hospital, Herning, Denmark; Fundacion Plagbol, La Paz, Bolivia; Unit for Health Promotion Research, Institute of Public Health, University of Southern Denmark, Odense, Denmark; Department of International Health, Immunology and Microbiology, University of Copenhagen, Copenhagen, Denmark

**Keywords:** Pesticide poisoning, Farmers Field School, IPM, Sustainability, Bolivia

## Abstract

**Background:**

Pesticide consumption is increasing in Bolivia as well as pest resistance, pesticide poisonings and pollution of the environment. This survey evaluates the training of small holder farmers on pesticide handling and ecological alternatives to reduce the negative pesticide effects.

**Method:**

A baseline survey was performed in 2002 and follow-up surveys in 2004 and 2009. Farmers were selected and trained on Integrated Pest Management (IPM) from 2002 to 2004 in Farmer Field Schools (FFS). After exclusions and drop outs, 23 FFS trained farmers could be compared to 47 neighbor farmers for changes in ‘knowledge, attitude and practice’ (KAP) on IPM and symptoms of poisoning when handling pesticides. Statistical analysis was performed with SPSS version 21.0 using χ^2^-test, Cochran’s Q test and Student’s T-test.

**Results:**

Improvements were seen in both groups but most significant among the FFS farmers. At baseline no difference were seen between the two groups apart from a more frequent use of personal protection among the FFS farmers. After the training was finished significant differences were seen between FFS farmers and neighbor farmers on all KAP variables, a difference reduced to six of the KAP variables in 2009. No difference was seen in self-reported poisonings after pesticide handling. FFS farmers improved their KAP scores markedly during training and there after retained their knowledge, while neighbor farmers improved during the entire period. Ecological farming without the use of pesticides increased most among the FFS farmers.

**Conclusion:**

The study showed a sustained improvement among Farmers Field School trained farmers on personal protection and hygiene when handling pesticides, knowledge and use of IPM and ecological alternatives and a reduction in self-reported symptoms after pesticide handling. Similar though less pronounced improvements was seen among neighbor farmers having had less training and information on pesticide handling and alternatives than the FFS trained farmers. Training of farmers on IPM and good agricultural practices has positive effects, but is scarce in Bolivia as in most low-income countries and must be encouraged to support an improved and sustainable food production and to protect the health of farmers and consumers as well as the environment.

## Introduction

Pesticide consumption in low-income countries is rapidly increasing, as well as pest resistance, acute pesticide poisonings and environmental pollution due to improper and unsafe handling. To address this Farmers Field Schools (FFS) with training of small holder rice farmers on Integrated Pest Management (IPM), were introduced by the United Nations Food and Agricultural Organization (FAO) in Asia around 1990 [1–3]. Later the FFS were spread to other parts of the world, and to include other types of crops, livestock, health issues, water and sanitation and democracy [4–7]. The FFS concept promotes local solutions to local problems and uses participatory adult training processes and ‘learning by doing’ in the fields [1–3]. IPM is not uniformly defined but most often emphasizes the growth of a healthy crop with minimal disruption to agro-ecosystems [2, 3, 8]. IPM encourages natural pest control mechanisms keeping pesticides and other interventions to reasonable economic levels while reducing health and environmental risks [2, 3, 8]. The FFS concept have shown promising results among trained farmers most often by increasing yields and reducing pesticide use [1–7, 9–17]. Some surveys have pointed to a possible broader effect by empowering participants improving their ability to plan, organize, take leadership and realize collective experiments [1–3]. Some studies have also focused on health and environmental outcomes when reducing and improving pesticide use and handling [1, 4, 14, 15].

In Bolivia pesticide use has tripled over the last decade leading to a growing problem of acute poisonings due to accidents, suicides, and improper handling in agriculture and public health vector control programs [18–20]. One study showed improved technical handling of pests in potatoes after training farmers in FFS, on village workshops and through short messages in the radio [21].

This survey presents the effects of training a cohort of small holder farmers from 2002 to 2004 with follow-up studies in 2004 and 2009. The objective was to show if FFS training would have long term impact on farmers knowledge, attitude and practice (KAP) to improve the handling of pesticides using IPM strategies and to lower the number self-reported symptoms of acute poisoning after pesticide handling.

## Methods

### Study area and population

The Plagbol project was launched in 2001 and continued until 2013 promoting training, information and awareness rising among farmers, health care workers, teachers and pre-graduates to prevent pesticide poisonings and environmental pollution. The training activities of the first project phase from 2001 to 2004 were implemented in four municipalities within the La Paz County. This is an area with varying climates, from temperate to subtropical, making it possible to grow a wide variety of crops. Most pesticide spraying takes place from October to May.

Farmers Field School training was offered to 48 hamlets/small villages known to have a significant use of pesticides and good accessibility by road or river with a total population of around 10.000 people (pers com Plagbol Project). Local authorities and farmers were extensively involved in the selection of hamlets, selection of the farmers for FFS training and later planning and execution of the trainings to create local ownership and improve sustainability of the interventions.

Criteria taken into consideration in the selection of the farmers were ‘a person of confidence’, ‘ability to read and write’ and ‘permanent residence in the hamlets’ to maintain the trained human resources in the area. FFS trainings took place in the different hamlets to enable the rest of the villagers to follow the courses when they took place in their hamlet.

### Intervention

The FFS farmers were trained in IPM methods to improve their Knowledge Attitude and Practice (KAP) concerning pesticide handling and ecological farming methods during 14 theoretical and practical courses of one to two days duration. After having completed at least 12 of the courses the farmers were given a diploma as an FFS farmer. The intensive training courses took place over a period of 20 month from June 2002 to February 2004. Educational booklets for the seven theoretical modules were developed by the project agronomist and doctor on: 1. Pedagogic, 2. The World of Pesticides, 3. The Use of Pesticides, 4. Agricultural Pests, 5. Health Effects of Pesticides, 6. IPM Methods and 7. IPM in Tomatoes [22]. A draft version of the booklets was used for each training course and then modified according to the input from the farmers and project supporters before been printed in a final version for distribution among the farmers.

A minimum of two courses on ‘Integrated Pest Management’ and ‘Adequate use of pesticides’, were undertaken in the hamlets by the FFS farmers to train their neighbor farmers as well as informal knowledge sharing taking place on a day to day basis. To facilitate dissemination from FFS farmers to neighbor farmers the first module was on pedagogy. During the FFS training the farmers produced their own teaching material such as flipcharts, herbarium and insect collections to be used for teaching sessions in their hamlets, and rehearsed by teaching each other.

To improve awareness in the general population, radio and television programs were transmitted and informative pamphlets, folders and copies of the training materials were distributed through the internet and as hard copies.

### Study design

A baseline survey was conducted among 201 farmers from March to April 2002 before the selection of the farmers to go for FFS trainings took place. From this baseline 40 FFS farmers, out of 60 FFS trained farmers, could be identified and included in the first follow-up survey taking place from October to November 2004. It was decided to include twice as many neighbor farmers from the baseline study. They were invited at meetings in the hamlets and via ‘mouth to mouth method’ to show up at a central place in the hamlets at a given date and time. Eighty nine neighbor farmers showed up and were interviewed in their villages together with the FFS farmers.

**Figure 1:**
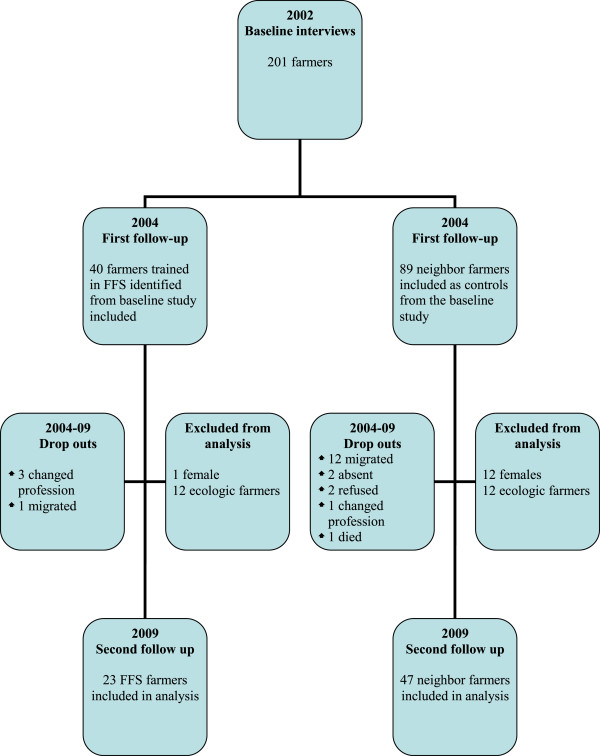
**Flow chart of study participants.**

Due to a very skewed gender distribution in the two groups of farmers it was decided to exclude female farmers from the main analysis to avoid gender bias as a former study did show significant differences between Bolivian male and female farmers regarding pesticide handling and symptoms [23]. The farmers who shifted to ecological farming were also excluded from the main analysis as most of the questions about classes of pesticides used, personal protection and hygiene while handling pesticides and symptoms of pesticide intoxications were irrelevant to this group. Then there were some drop outs mainly due to migration. We ended up with 23 FFS farmers and 47 neighbor farmers with a full data set for the main analysis comparing data from 2002, 2004 and 2009, see flow chart Figure 1.

The survey was approved by the Medical Ethical Committee of Bolivia, and all participants signed an informed consent form before the interviews were conducted.

The survey was based on a pre-tested questionnaire developed for the 2002 baseline study using interview forms developed in former studies from Bolivia, Denmark and the US [24]. The questionnaire consisted of closed and open-ended questions, including age, sex, education, size of cultivated land, crops cultivated, pesticides and alternatives used, knowledge, attitudes and practice when buying, handling and storing pesticides; perceived negative impact from pesticides; and own experience with poisoning after handling pesticides. The interviews were conducted by trained Spanish speaking health care workers, agronomists and students. The follow-up surveys compared changes within and between the two groups of farmers.

The outcome variables are all dichotomous variables. The variable ‘use of WHO class I pesticide’ was elaborated from a question about which pesticides the farmers were using and then the pesticides were categorized into the different WHO toxicity classes.

Three of the variables analyzed are aggregated variables with each variable included given equal weight and then dichotomized. The use of aggregated variables was preferred to be able to present as much information as possible in the analysis, and one can argue that aggregated variables may provide a better overall picture of the type of exposure and the association with outcome, resulting in a more robust analysis.

The variable ‘Personal protection’ was aggregated from the variables ‘using long sleeved shirts when spraying’, ‘using long trousers when spraying’, ‘using a hat when spraying’, ‘using a mask when spraying’, ‘using gloves when spraying’, ‘using boots when spraying’, ‘washing body after spraying’, ‘changing clothes after spraying’, and ‘refraining from eating, chewing coca or smoking while spraying’. The aggregated variable was dichotomized according to positive answers to at least 6 of the 9 variables.

The variable ‘Good technical handling’ was aggregated from the variables ‘adjusting sprayer before spraying’, ‘washing sprayer after spraying’, ‘refraining from spraying same day as harvest’, ‘no re-entry into the field the same day as spraying’, ‘burning/burying empty pesticide containers’, ‘storing pesticides locked up’. The variable was dichotomized according to positive answers to at least 4 of the 6 variables.

The variable ‘Knowledge of pesticide toxicity’ was aggregated from the variables ‘do you think pesticides can have negative effects on human health’, ‘do you think pesticides can have negative effects on animal health’, ‘do you think pesticides can have negative effects on the environment’, ‘can you mention two or more symptoms of pesticide poisoning’, ‘knowing that red color on pesticide container means highest toxicity’ and ‘knowing that green color on pesticide container means low toxicity’. The variable was dichotomized according to correct answers to at least 5 of the 6 variables.

To give an overview of performance in the two groups and its development through the years 2002, 2004 and 2009 an error bar graph with 95% CI was elaborated calculating a mean KAP score for each year. The KAP score was created by aggregating all of the 27 KAP variables and giving all variables the same weight the maximum KAP score was 27.

### Data analysis

The non-parametrical Cochran’s Q test for k related samples were used for changes in KAP-variables and symptoms within each group of farmers over the whole period 2002 to 2009.

Mc’Nemar’s test for paired samples was used to compare difference within each farmers group in the two periods 2002–04 and 2004–09.

χ^2^ test were used to compare differences between the two groups of farmers at baseline and at each follow up.

T-test were used for calculating age, years in farming, size of land cultivated and for calculating mean KAP-score with 95% CI.

Missing values were kept missing. The analysis was conducted with SPSS version 21.0.

## Results

### General data

A significant difference between the two groups was found for age and years working as a farmer (Table 1). FFS farmers cultivated more land and were better educated than their neighbor farmers, although these differences were not significant. Comparing age and farming years it was seen that the typical time for starting to farm as a profession was around 16 years of age.

**Table 1:** **General data at baseline 2002 among FFS farmers (N = 23) and neighbor farmers (N = 47)**

Variables		Mean	Range	p-value
Age, mean	FFS farmers	34,6	22-61	0.01
	Neighbor farmers	42,6	19- 70	
Years in farming, mean	FFS farmers	19,1	1-40	0.03
	Neighbor farmers	26,2	3-60	
Hectares grown, mean	FFS farmers	2,1	0,2-15,1	0.06
	Neighbor farmers	1,1	0-4,5	
		%		
Farming in temperate climate	FFS farmers	65,2		0.81
	Neighbor farmers	68,1		
Educational level above primary school	FFS farmers	73,9		0.08
	Neighbor farmers	52,2		
Received course on pesticide handling	FFS farmers	26,1		0.51
	Neighbor farmers	19,1		

χ^2^-test and Student’s T-test used for calculating p-values.

Comparing participating farmers with excluded farmers and drop outs no significant differences were found between the two groups on the general variables, KAP variables or symptoms of poisoning after spraying.

### Effect of the intervention within FFS farmers and neighbor farmers

Analyzing the changes from 2002 to 2009 within each of the two groups of farmers with Cochran’s test all variables improved significantly among the FFS farmers, while 6 significant improvements and one borderline improvement were seen in the group of neighbor farmers (Table 2).

**Table 2:** **KAP variables and symptoms of intoxication among FFS farmers and neighbor farmers from 2002-04-09**

Categories/Variables	Farmer groups	2002 χ ^2^-test		2004 χ ^2^-test		2009 χ ^2^-test		2002-04-09 Cochran’s test
		N (%)	p-value	N (%)	p-value	N (%)	p-value	p-value
No use of WHO class I pesticides	FFS farmers	3/23 (13)	0.67	16/23 (69.6)	0.00	17/23 (73.9)	0.07	0.00
	Neighbor Farmers	8/47 (17)		12/47 (25.5)		24/47 (51.1)		0.00
Have sprayed less than three times past month	FFS farmers	12/23 (52.2)	0.10	18/23 (78.3)	0.00	16/23 (69.6)	0.07	0.04
	Neighbor Farmers	15/47 (31.9)		17/23 (36.2)		22/47 (46.8)		0.28
Think pesticide use can be lowered without affecting harvest	FFS farmers	7/23 (30.4)	0.70	20/23 (87)	0.00	17/23 (73.9)	0.01	0.00
	Neighbor Farmers	12/46 (26.1)		16/47 (34)		20/47 (42.6)		0.27
Knows alternative methods to pesticide use	FFS farmers	4/23 (17.4)	0.43	22/23 (95,7)	0.00	23/23 (100)	0.00	0.00
	Neighbor Farmers	5/47 (10.6)		10/47 (21,3)		15/47 (31.9)		0.22
Reads instructions on pesticide container before use	FFS farmers	7/23 (30.4)	0.85	23/23 (100)	0.01	22/23 (95.7)	0.26	0.00
	Neighbor Farmers	13/46 (28.3)		34/46 (73.9)		40/46 (87.0)		0.00
Refrain from blowing spray-head when obstructed	FFS farmers	12/23 (52.2)	0.47	23/23 (100)	0.00	21/22 (95.5)	0.04	0.00
	Neighbor Farmers	18/42 (42.9)		33/47 (70.2)		35/47 (74.5)		0.00
‘Good personal protection’ (aggregated variable)	FFS farmers	8/23 (34.8)	0.03	19/22 (86.4)	0.00	18/21 (85.7)	0.00	0.00
	Neighbor Farmers	6/46 (13.0)		6/47 (13)		20/45 (44.4)		0.00
’Good technical handling’ (aggregated variable)	FFS farmers	5/22 (22.7)	0.63	19/22 (86.4)	0.00	16/20 (80)	0.00	0.00
	Neighbor Farmers	8/45 (17.8)		21/42 (50)		15/41 (36.6)		0.02
‘Good knowledge of pesticide toxicity’ (aggregated variable)	FFS farmers	7/23 (30.4)	0.31	22/23 (95.7)	0.00	21/23 (91.3)	0.00	0.00
	Neighbor Farmers	9/46 (19.6)		21/47 (44.7)		18/41 (43.9)		0.06
No self-reported symptoms after spraying past year	FFS farmers	6/23 (26.1)	1.00	14/23 (60,9)	0.54	16/22 (72.7)	0.15	0.00
	Neighbor Farmers	12/46 (26.1)		25/47 (53,2)		25/46 (54.3)		0.02
No self-reported symptoms after spraying past month	FFS farmers	12/23 (52.2)	0.61	20/23 (87)	0.31	17/22 (77.3)	0.43	0.01
	Neighbor Farmers	27/46 (58.7)		36/47 (76.6)		32/47 (68,1)		0.12

(χ^2^-test used for calculating significant differences between the two farmers groups and Cochran’s Q test for calculating significant differences within each farmers group).

**Figure 2:**
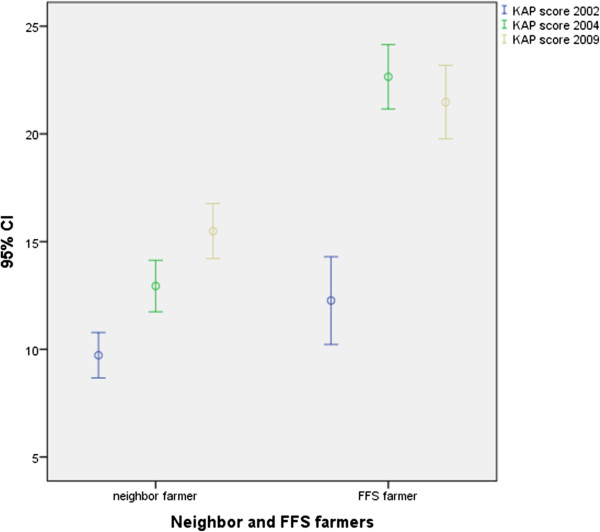
**Changes in KAP score of FFS farmers (N = 23) and neighbor farmers (N = 47) from 2002 to 2004 to 2009, mean values with 95% CI, (maximum score = 27).**

Analyzing each of the two periods 2002–04 and 2004–09 the FFS farmers had improved by far the most at the first follow up in 2004 where Mc’Nemar’s test for paired data showed significant improvements (p < 0.05) in 10 of the 11 variables with the exception being the variable on ‘number of times sprayed past month’. The neighbor farmers showed improvement in 6 of the 11 variables including all ‘personal security measures’ and ‘pesticide toxicity and intoxication’ variables except for the variable on good personal protection (p < 0.05). From 2004 to 2009 no significant changes were seen among the FFS farmers, while the neighbor farmers still improved significantly, as the variables ‘No use of pesticide WHO class 1’, ‘Knowledge of alternatives to pesticide use’ and ‘Good personal protection when handling pesticides’ became significant (p < 0.05), while the variables ‘Good technical handling’ and Good knowledge of pesticide toxicity’ became borderline significant (p < 0.10) and ‘No self-reported symptoms after spraying past month became non-significant (p > 0.05) changing from being significant at first follow up.In Figure 2 the change is illustrated in a graph showing that the FFS farmers almost doubled their mean KAP-score during their intensive training period from 2002 to 2004 with no further improvement there after while the neighbor farmers showed a steadier but less pronounced improvement through the whole period from 2002 to 2009.

### Comparison of the intervention effect between the FFS farmers and the neighbor farmers

At baseline in 2002 only the aggregated variable ‘good personal protection’ showed a significant difference between the FFS farmers and neighbor farmers (χ^2^-test, p < 0.05), see Table 1. In 2004 the χ^2^-test showed significant differences between the two groups in all the KAP variables, and in 2009 the number of variables with significant differences between the two groups was reduced from nine to six, (Table 2).

To evaluate a possible influence on KAP variables by age, education, years in farming, size of land cultivated and climate a stratified analysis was done. The only significant findings were that farmers from the subtropical climate showed a better performance on the variables ‘Refrain from blowing nozzle when obstructed’, ‘Good personal protection’, ’Good technical handling’ and ‘Good knowledge of pesticide toxicity’. This might be explained by the finding that the farmers who had received courses on pesticide handling from pesticide retailers and others before project start in 2002 were primarily farmers living in the subtropical versus the temperate climate (p < 0.05).

### Ecological farming

Twelve of the initial 40 FFS farmers (30%) and 12 of 89 neighbor farmers (13.5%) changed to ecological farming (χ^2^-test p < 0.00). All FFS farmers improved their knowledge on alternative ecological farming methods with an increase in the mean number of mentioned pest controlling methods from 0.3 to 2.4 per farmer, compared to the conventional farmers, 0.2 to 0.4 methods per farmer. The methods reported were light and color traps for attracting and killing insects and different plant extracts used for making natural pesticides for spraying on the crops.

Farmers who tried to practice ecological farming were more likely to be farmers from the subtropical climate 23/56 (41.1%) compared to 7/73 (9.6%) of the farmers from the temperate climate (χ^2^-test p < 0.00).

## Discussion

The survey showed significant improvement in pesticide handling and use of alternatives to pesticides among farmers trained at farmer’s field schools, an improvement that was maintained 5 years after the training stopped. The same though less profound was seen among the neighbor farmers, which could be due to dissemination of knowledge from FFS trained farmers in combination with diffusion of information into society by the Plagbol project. It could also simply be due to an improved knowledge level on IPM and good agricultural practices among Bolivian farmers in general. The intensively trained FFS farmers improved during their training period from 2002–04, while the neighbor farmers improved over the whole period, and never reached the higher ‘KAP-score’ of the FFS farmers, a difference probably reflecting the different training and information level in the two groups. A considerable number of farmers turned to ecological farming thereby reducing the negative effects on health and environment by pesticide use. The results must be interpreted with caution due to the limited number of participants and the lack of a control group without influence by the project interventions.

In the selection of farmers we saw that farmers tended to select the better educated and younger men to go for trainings. This experience must be taken into account when starting similar FFS trainings with the aim to include more women and resource poorer farmers in the trainings. The selection of literate farmers was though promoted by the project to improve the chances of having a positive effect of the trainings. This has also been seen in other studies showing skewed age, education, social level and gender distribution among the FFS participants [6, 7, 12, 25].

An improved pesticide handling and use of IPM methods among FFS trained farmers has been shown in several other studies [1–4, 6, 7, 9, 12, 13, 15–17, 21, 25, 26]. Some studies found the acquired knowledge and positive results were retained, although evaluated over a shorter time period than in the actual study [1, 4, 6, 12]. This is though questioned by others finding some of the positive results were lost over time [11]. Supporting possible sustainability of the FFS trained farmers is the finding in a later evaluation of the Plagbol project from 2012 that FFS farmers are now recognized as specialists on crop protection and hired for training of farmers in other hamlets/villages by their Municipalities 8 years after their training have stopped [27].

Whether or not positive experiences in one place can be transferred to different crops, farming systems and cultures is debated and there is little doubt about the need for adaptation of the FFS concept and IPM training to local circumstances if success shall be expected [1, 3, 4, 6, 28, 29]. An evaluation from 2013 in the Plagbol Project indeed points to this as farmers in Focus Group discussions mentioned ´that growing special crops like coffee and tee favored IPM and ecological farming as a demand for ecological products made the prices increase compared to the conventionally grown [30].

Our finding of a reduced number of farmers reporting the use of WHO class I pesticides could be a reflection of what is seen in other studies showing a significant reduction in pesticide use after FFS training [1–7, 9–17, 25]. The increase in yields shown in these studies as well is not necessarily linked to the reduction in pesticide use, but might as well be due to the ‘good agricultural practices’ taught alongside the use of alternatives to pesticides when teaching IPM in FFS, and as some pointed out IPM teaching should be renamed ICM (integrated crop management) as pest control implies a lot of other cultural practices apart from a correct and minimized pesticide handling and use [28].

The improved use of personal protective equipment (PPE) and hygiene have been seen in other intervention studies among FFS trained farmers as well [6, 9, 15, 31]. A problem regarding the use of PPE in most low income countries is that good PPE is scarce, expensive and not comfortable to wear under hot tropical conditions [32]. A solution could be to focus on the cheapest, most simple and effective PPE measures like the use of gumboots, gloves and changing and washing long sleeved pant and shirt after spraying.

An important finding is the reduction in the number of farmers reporting poisoning symptoms after pesticide spraying which might be related to the improvement seen in the KAP variables, and especially in the two variables ‘reading instructions for use’ and ‘refrain from blowing spray nozzle when obstructed’, as they have been found to be independent risk factors for self-reported symptoms of pesticide poisoning and Acetylcholine-esterase depression [18]. The reduction of symptoms after spraying has been evaluated in other studies where an increase in the use of IPM methods and personal protection when handling pesticides seems to have resulted in fewer symptoms of poisoning and affection of the blood Acetyl Cholinesterase level [1, 4, 14, 15].

Most often the economic aspect has been evaluated as an argument for adoption of IPM but to include health and environmental aspects as arguments for the adoption and diffusion of IPM is a possibility that should be explored. Farmers mention the importance of these aspects, not only the economical one when deciding whether or not to shift to IPM farming or ecological farming [30].

### Weaknesses of the survey

The size of the study is a limitation, and made it difficult to use a controlled analysis due to the broad confidence intervals coming up. The farmers who participated in the trainings in the FFS were a selected group being younger and better educated than their neighbor farmers whom we used for comparison. A random selection of FFS farmers was not possible as the farmers selected among their own representatives to go for FFS training. For comparison of the effects of the training within the same group of farmers this was not a problem as the farmers were their own controls. When comparing the changes between the two groups an analysis controlling for age and education would have been desirable to minimize the possibility for confounding, although age and education were of no significance when analyzing KAP variables at baseline in 2002. Random selection is difficult to practice in most low-income countries as no updated population registers exists, most people are functional illiterate and a formal direction with road name and number to send mail to are not available. Neighbor farmers were therefore invited by direct oral communication at village meetings or if found at home when visiting the villages.

The use of self-reported symptoms when spraying pesticides might introduce recall bias as they are nonspecific and people might have difficulty in recalling them a whole year or even a month previously. Some groups with increased awareness (FFS farmers) and with major events (very sever poisoning episodes) might have longer recall than other groups and events.

A difference in climate and pest pressure at the different times of the data gathering is a problem influencing the number of sprayings and the chances of getting poisoned past month and must be taken into account when interpreting changes in these variables.

Studying information dissemination between farmers and their neighbors was not possible due to small numbers and lack of a control group without influences from the project interventions. In a future study including a control group or a possible network analysis exploring social capital dimensions and the use of mixed methods could be more appropriate to explore dissemination of knowledge as shown by others [9].

## Conclusion

The study showed a sustained improvement among Farmers Field School trained Bolivian farmers on personal protection and hygiene when handling pesticides, knowledge and use of IPM and ecological alternatives and a reduction in self-reported symptoms after pesticide handling. Similar though less pronounced improvements was seen among neighbor farmers having had less training and information on pesticide handling and alternatives than the FFS trained farmers.

Training of farmers on IPM and good agricultural practices has positive effects, but is scarce in Bolivia as in most low-income countries and must be encouraged to support an improved and sustainable food production and to protect the health of farmers and consumers as well as the environment.
